# Genetically Encoded Calcium Indicators Can Impair Dendrite Growth of Cortical Neurons

**DOI:** 10.3389/fncel.2020.570596

**Published:** 2020-10-20

**Authors:** Ina Gasterstädt, Alexander Jack, Tobias Stahlhut, Lisa-Marie Rennau, Steffen Gonda, Petra Wahle

**Affiliations:** Developmental Neurobiology, Faculty of Biology and Biotechnology, Ruhr University Bochum, Bochum, Germany

**Keywords:** postnatal development, dendritogenesis, neurite growth, dendritic injury, cell death, Fe65, calcium imaging

## Abstract

A battery of genetically encoded calcium indicators (GECIs) with different binding kinetics and calcium affinities was developed over the recent years to permit long-term calcium imaging. GECIs are calcium buffers and therefore, expression of GECIs may interfere with calcium homeostasis and signaling pathways important for neuronal differentiation and survival. Our objective was to investigate if the biolistically induced expression of five commonly used GECIs at two postnatal time points (days 14 and 22–25) could affect the morphological maturation of cortical neurons in organotypic slice cultures of rat visual cortex. Expression of GCaMP3 in both time windows, and of GCaMP5G and TN-XXL in the later time window impaired apical and /or basal dendrite growth of pyramidal neurons. With time, the proportion of GECI transfectants with nuclear filling increased, but an only prolonged expression of TN-XXL caused higher levels of neurodegeneration. In multipolar interneurons, only GCaMP3 evoked a transient growth delay during the early time window. GCaMP6m and GCaMP6m-X_C_ were quite “neuron-friendly.” Since growth-impaired neurons might not have the physiological responses typical of age-matched wildtype neurons the results obtained after prolonged developmental expression of certain GECIs might need to be interpreted with caution.

## Introduction

In eukaryotic cells, calcium controls virtually all fundamental processes including motility, metabolism, secretion, gene transcription, and finally even cell death. Calcium is of particular importance for the proper development and functioning of the nervous system. Crucial functions such as migration, morphofunctional development, synaptic transmission up to learning, and memory consolidation require a precise spatial and temporal regulation of calcium signals. Neural cells have evolved an intricate and complex toolkit to process calcium-related signals (for review, Brini et al., 2014). In resting conditions, the intracellular calcium concentration of neurons is low between 50 and 100 nM. Activity causes the levels to increase up to 100-fold (for review, Berridge et al., 2000) with so-called calcium blips, puffs, and waves occurring in all compartments of a neuron. Calcium transients are induced by ionotropic and metabotropic signaling and are mediated by voltage-, ligand-, or store-operated channels at the cell membrane or in internal organelles (Bengtson and Bading, 2012). A battery of endogenous and partly cell class-specific calcium-binding proteins serve as cytosolic calcium buffers and are of particular importance for the kinetics of calcium signals (Schwaller, 2010).

Monitoring cellular calcium dynamics has become of fundamental interest in neuroscience. Two major groups of calcium indicators are commonly used. Chemical calcium indicators such as fura-2, fluo-4, or Oregon Green 488 BAPTA-1 consist of a calcium chelator and a fluorophore which changes fluorescence upon calcium binding. These chemical calcium sensors are commonly delivered into single cells by sharp microelectrodes or patch-clamp pipettes or to larger cell populations by injections (Stosiek et al., 2003; Grienberger and Konnerth, 2012), or non-invasively (Hamad et al., 2015). Calcium dyes are not suitable for long term imaging experiments; thus, GECIs are preferred, either as FRET-based (Miyawaki et al., 1997; Heim et al., 2007; Mank et al., 2008) or single-fluorophore sensors (GCaMP family; Nakai et al., 2001; Tian et al., 2009). GCaMPs, which are commonly used as effective single-fluorophore GECIs, consist of circular permutated EGFP and a calmodulin calcium-sensing domain with four calcium ion binding sites (Nakai et al., 2001). To date, a battery of GECIs is available with differences in signal-to-noise ratio, calcium affinity, response kinetic (Grienberger and Konnerth, 2012; for reviews, Rose et al., 2014). GECI expression is accomplished *via* *in utero* electroporation, viral transduction, transgenic approaches, or gene-gun transfection, depending on the experimental system. GECIs are the tool of choice for cell-specific expression (Bozza et al., 2004) and long-term calcium imaging (Holtmaat et al., 2009; Andermann et al., 2010; Chang et al., 2017) from somata, dendrites, and axons (Sadakane et al., 2015; Inoue et al., 2019; Dana et al., 2019).

Despite the numerous advantages, GECIs act as calcium buffers (McMahon and Jackson, 2018). Expression in long-term applications has been reported to have potentially harmful effects on cellular calcium homeostasis. For instance, prolonged-expression of *in utero* electroporated GCaMP3 can cause its nuclear accumulation at postnatal days 25–28 in a subset of the targeted neurons (Tian et al., 2009). Such nuclear-filled neurons display reduced activity-evoked calcium changes and prolonged decay times of calcium signals suggesting altered physiology. Further, long-term high-level expression of GCaMPs has been reported to result in punctate clusters of GCaMPs and non-functional calcium sensor protein possibly by interaction with other cellular components, although no gross abnormalities at the cellular level have been observed (Hasan et al., 2004). Other studies show that prolonged GCaMP expression can impair morphological development in dissociated cortical neurons (Yang et al., 2018) or leads to episodes of hyperexcitability (Steinmetz et al., 2017). In cortical tissue expressing GCaMPs *via* viral infection, a certain proportion of neurons acquires nuclear fluorescence which correlates with abnormal physiological responses, although this seems to not disturb the cortical circuits in general (Chen et al., 2013). The nuclear GECI accumulation can progress to a loss of dynamic calcium signaling and eventually to apoptosis (Tian et al., 2009; Grienberger and Konnerth, 2012). Nuclear calcium signaling (Bading, 2013) has been shown to regulate the genetic program for neuroprotection (Zhang et al., 2009) and nuclear calcium buffering is important for shaping the dendritic morphology (Mauceri et al., 2011, 2015). Whether nuclear accumulation is a cause for or the consequence of the initiation of neuropathological processes is unknown. For cytosolic GECIs, the undesired side-effects have been reported to be due to the calmodulin domain of the GECI directly interfering with the gating of endogenous L-type Ca_V_1.3 calcium channel subunits and their interaction with endogenous calmodulin, eventually disrupting the essential excitation-transcription signaling, and triggering apoptosis beginning at DIV 10 in dissociated neurons (Yang et al., 2018). The newly developed sensor GCaMP6m-X_C_ has been reported to no longer interfere with endogenous Ca_V_1/CaM interaction because the actively interacting group is shielded by an additional apoCaM binding motif (Yang et al., 2018).

Recent understanding is that a variety of endogenous calcium sensors of the EF-hand family members naturally interact with neurotransmitter receptors. For instance, CB1R (Angelats et al., 2018) and NMDA receptors (Franco et al., 2018) interact with the calmodulin, calneuron-1, and NCS1. These interactions are required for successful CB1R-mediated activation of G-protein signaling (Angelats et al., 2018). In mouse models of neurodegenerative disorders, the misregulation of the NMDAR-NSC1 complex was observed in neurons and microglia cells leading to a blunted MAP kinase activation. It is unknown if GECIs could somehow interfere with such interactions. Calcium signaling mediated by ionotropic glutamate receptors (iGluRs) such as NMDA, AMPA, and kainate receptors (Hamad et al., 2011, 2014; Jack et al., 2018), as well as voltage-gated calcium channels, is important for dendritic elongation and branching, pruning and self-avoidance, and spine development during early postnatal dendritic maturation (Ledda and Paratcha, 2017). Calcium signaling *via* CaM and CaM kinases I, II, and IV and cAMP response element-binding protein evoke dendritic growth as well as pruning (Redmond, 2008; Uhlén et al., 2015; Valnegri et al., 2015). Therefore, we selected TN-XXL, GCaMP3, GCaMP5G, GCaMP6m (the *K*_D_ values for binding calcium vary in that order from highest to lowest; Rose et al., 2014) and GCaMP6m-X_C_ because of its shielded Cam domain. Employing organotypic slice cultures we aimed to investigate whether the prolonged-expression of these GECIs in two postnatal time windows could affect the morphological maturation of cortical pyramidal cells and multipolar interneurons.

## Materials and Methods

### Organotypic Cultures (OTCs)

Visual cortex Organotypic cultures (OTCs) were prepared from P0/P1 neonate rat pups (Long Evans) as described (Hamad et al., 2014). Briefly, cortex blocks were cut into 350 μm slices with a McIlwain tissue chopper (Ted Pella, Redding, CA, USA). Slices were mounted on a coverslip with a plasma/thrombin coagulate and cultured at 37°C in roller-tubes with 700 μl semi-artificial medium containing: 25% adult horse serum, 25% Hank’s balanced Salt Solution, 50% Eagle’s Basal Medium, 1% NeuroCult™ SM1 Neuronal Supplement (STEMCELL Technologies, Cologne, Germany, Cat.# 05711), 1 mM L-Glutamine (all from Life Technologies, Karlsruhe, Germany), and 0.65% D-Glucose (Merck, Darmstadt, Germany). Excessive glial growth was prevented by treating OTCs at DIV 2 with a mix of uridine, cytosine-ß-D-arabinofuranoside, and 5-fluorodeoxyuridine (all from Sigma–Aldrich, Steinheim, Germany) for 24 h. The medium was changed every third day. OTCs from every individual animal (4–5 animals per batch) were allocated to all experimental conditions run with this batch of cultures.

### Plasmids

All plasmids (Table 1) were prepared as endotoxin-free solutions using the EndoFree Plasmid Maxi Kit (Qiagen, Hilden, Germany, Cat.# 12362). Plasmid stocks were diluted to 1 μg/μl and stored at −20°C.

**Table 1:** Plasmids.

Plasmid	Promoter	Source	Catalog number
pEGFP-N1	CMV	Clontech, Heidelberg, Germany	Cat.# 632370
pmCherry-N1	CMV	Clontech, Heidelberg, Germany	Cat.# 632523
G-CaMP3	CMV	Tian et al. (2009); gift from Loren Looger	RRID:Addgene_22692
pCMV-GCaMP5G	CMV	Akerboom et al. (2012); gift from Douglas Kim and Loren Looger	RRID:Addgene_31788
pGP-CMV-GCaMP6m	CMV	Chen et al. (2013); gift from Douglas Kim	RRID:Addgene_40754
pN1-GCaMP6m-X_C_	CMV	Yang et al. (2018); gift from Xiaodong Liu	RRID:Addgene_111543
TN-XXL pcDNA3	CMV	Mank et al. (2008); gift from Oliver Griesbeck	RRID:Addgene_45797
Fe65-EGFP	CMV	Minopoli et al. (2001); gift from Thorsten Müller; developed by Dr. Tomasso Russo, Naples University, Italy	n.a.

### Biolistic Transfection and Immunohistochemistry

Transfection was carried out as described (Hamad et al., 2014; Jack et al., 2018). Briefly, cartridges were prepared by coating 7 mg gold microparticles (1 μm diameter; Bio-Rad Laboratories, Feldkirchen, Germany) with 10 μg plasmid encoding EGFP or one of the GECIs indicated. Cultures were blasted at DIV3–4 (Helios Gene Gun, Bio-Rad Laboratories, Feldkirchen, Germany) with 180 psi helium pressure and continued to DIV 14 or DIV 22–25. For one assessment, cultures were transfected at DIV 20 and continued to DIV 30. Then, as described (Hamad et al., 2014; Jack et al., 2018), OTCs were fixed with 37°C 4% paraformaldehyde in 0.1 M phosphate buffer pH 7.4 for 2 h, rinsed, permeabilized with Triton X-100 (0.3% in phosphate buffer for 30 min), blocked with TBS/BSA solution, and incubated in mouse anti-GFP antibody (1:1,000; clone GSN24, Sigma–Aldrich, Steinheim, Germany, RRID:AB_563117) overnight. The antibody reacts with the GFP moiety in GCaMPs and the FRET-based sensor TN-XXL and stains the neurons completely. Immunoperoxidase staining proceeded with biotinylated goat anti-mouse (1:1,000; Dako A/S, Glostrup, Denmark, Cat.# E043301-2) for 3 h, followed by ABC reagent for 2 h (Vector Laboratories Inc. Burlingame, CA, USA, Cat.# PK-7100, RRID:AB_2336827), an HRP reaction with 3,3′-diaminobenzidine (Sigma–Aldrich, Steinheim, Germany) and H_2_O_2_. The resulting DAB product was intensified for 30 s with 1% OsO_4_ (Sigma–Aldrich, Steinheim, Germany). Cultures were dehydrated and coverslipped in DEPEX (Sigma–Aldrich, Steinheim, Germany). Fe65-EGFP was cotransfected with mCherry as reported early at DIV 12, and nuclear spheres were detected by EGFP fluorescence in live neurons within 12–24 h. After ~50 h of expression, the transfectants were immunoperoxidase-stained with mouse anti-mCherry (1:1,000; Clontech, Heidelberg, Germany, Cat.# 632543) for neuronal reconstruction.

### Confocal Calcium Imaging

Neurons transfected with GCaMP6m were recorded to qualitatively document spontaneously occurring calcium events in neurons with translucent vs. occluded nuclei. Briefly, the culture was rinsed several times with oxygenated ACSF, and allowed to adjust to conditions for 1 h in the roller incubator. Spontaneous activity of the transfectants was recorded by imaging cultures with a Leica TCS SP5 confocal microscope (Leica, Mannheim, Germany) with a ×10 objective at 1,400 Hz and 3 frames/s as described (Hamad et al., 2014; Jack et al., 2018).

### Morphometry and Assessment of Neurodegeneration

For morphometrical analysis, immunoperoxidase-stained neurons were reconstructed with the Neurolucida system (MicroBrightField, Inc., Williston, VT, USA) by trained observers blinded to conditions. All reconstructions were crosschecked by a blinded observer for the correctness and to classify the cell type. Pyramidal cells and multipolar sparsely spinous interneurons were classified by criteria of dendritic and axonal patterns. Pyramidal neurons were grouped as follows: those of layers II/III have an apical dendrite reaching into layer I, and those of layers V/VI have an apical dendrite ending in middle layers; thick and thin tufted large layer V pyramidal cells occurred too rarely and were not sampled (Hamad et al., 2014; Jack et al., 2018). Care was taken to only reconstruct neurons with a clear translucent nucleus. Further, since GECIs expressing neurons may suffer from nuclear filling, we assessed the nuclear staining with fluorescent confocal images and the DAB stained material used for reconstruction.

**Figure 1:**
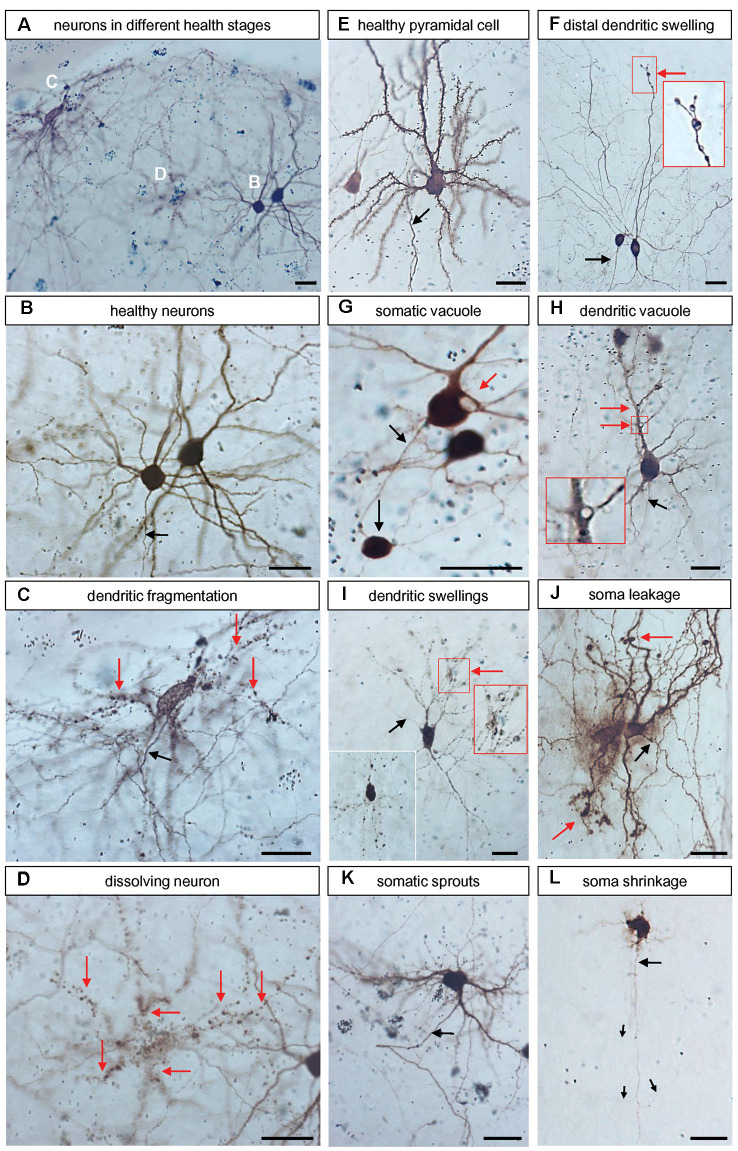
Representative examples of neurons with symptoms of degeneration. All microphotographs were taken from DIV 25 control with peroxidase immunostaining against EGFP. **(A)** Overview of four neurons in different stages of degeneration shown at higher magnification in **(B–D)** below. **(B)** Two healthy neurons, the axon is descending (arrow). **(C)** A neuron with dendrites breaking up into “rows of dots” (red arrows); note that the axon (black arrow) looks entirely healthy. **(D)** A cell in the final stage of dissolution; the argument that this has been a neuron derives from the observation that the rows of dots demarcate about six former dendritic trees (red arrows). **(E)** Healthy pyramidal neuron studded with spines and descending axons (arrow). **(F)** Two interneurons, one had distal dendritic swellings (red arrow, the boxed area is enlarged in the inset). **(G)** Neurons with a large somatic vacuole and a gigantic swelling at the axon’s first branch point which resembles a neuroma. **(H)** Quite healthy-looking pyramidal neuron with vacuoles at dendritic branch points (red arrow, boxed area enlarged). **(I)** A degenerating neuron with many swellings along the dendritic trees (red arrow, the boxed area is enlarged in the inset). A second inset at the lower left depicts other neurons where the dendrites are broken down into rows of stained dots, similar to **(C,D)**. **(J)** Neurons with leaky soma indicated by diffuse extracellular EGFP-like reaction product and dendritic swellings (red arrow). **(K)** Neurons with numerous fine short sprouts from the soma; note that the axon looked still unaffected (black arrow). **(L)** Neuron with shrunken soma and withering dendrites. The argument that this had been a pyramidal neuron derives from the observation that the axon (black arrow) is still looking unaffected (albeit very thin), it descended towards deeper layers and branches off three obliquely ascending collaterals (thin arrows). Pial surface to the top. The opaque or black (in focus) round gold particles occurred singly within the somata or clustered when accumulated by macrophages. Scale bars: 25 μm.

Further, with a ZEISS Axioskop equipped with a discussion bridge, we assessed with two trained observers blinded to conditions all neuronal transfectants in every slice culture for symptoms suggestive of neurodegeneration. The criteria are documented in Figure 1. We considered only neurons with recognizable axons that were completely labeled because dendritic injury often starts with distal dendritic blebbing. Proportions of neurons with degeneration were expressed in percent from all fully labeled neurons assessed per culture.

Further, confocal pictures of GECI fluorescence and nuclear DAPI fluorescence were taken, and the fluorescence intensity across soma and nucleus was determined per line scan with ImageJ; values are expressed as relative intensities.

### Statistical Analysis

Bar graphs, Sholl plots, and statistical analyses were done with Sigma Plot 12.3 (Systat Software, Erkrath, Germany). For all data, non-parametric ANOVA on ranks tests with corrections for multiple testing when appropriate (Bonferroni’s or Dunn’s test) were conducted followed by Mann–Whitney rank-sum tests. The number of independent preparations, cultures, and neurons analyzed is given in the graphs, tables, or the legends.

## Results

### Effects on Neurodegeneration

We have assessed the transfectants for symptoms of cellular degeneration in OTC transfected at DIV3/4 and stained at DIV 14. Figure 1 comprises a collection of such symptoms all photographed from control EGFP-transfected neurons. They belonged to two categories. First, dendritic blebbing, also known as dendritic injury, can be elicited for instance by excitatory neurotransmitters (Hamad et al., 2014; Jack et al., 2018) and can result in dendritic trees completely fragmented into “rows of dots” which remain immunoreactive for GFP. Second, somatic shrinkage can occur with the sprouting of numerous thin processes concurrent with a withering of the major dendrites. The dendritic retraction has been reported to proceed *via* distal thinning and fragmentation of dendritic tips (Khatri et al., 2018). For cells of both categories, the axonal morphology often remained surprisingly intact in our sample. Figure 1A and the higher magnifications in Figures 1B–D show three different stages: an entirely healthy neuron with axon and spiny dendrites (Figure 1B), a neuron with a healthy-looking axon and all dendrites fragmented into “rows of dots” (Figure 1C), and a cell with completely fragmented processes which was no longer safely recognizable as being neuronal (Figure 1D); such cells were excluded from the analysis. Another well-differentiated pyramidal neuron with spiny dendrites and a descending axon is presented in Figure 1E. Figure 1F shows two non-spiny interneurons, one of which had a dendrite with large distal swellings, it is enlarged in the inset. The neuron in Figure 1G had a large vacuole in the soma and the axon carried an enormous swelling at a branch point resembling a neuroma. Figure 1H shows a pyramidal cell with a large vacuole at a proximal dendritic branch point, it is enlarged in the inset. Figure 1I shows a neuron presenting with swellings along all dendritic branches, a cluster of swellings is enlarged in the inset to the right. The inset to the lower left shows a neuron that was even more advanced along the road to dendritic fragmentation. Figure 1J shows two cells with a massive halo of immunostained EGFP suggesting a leaky somatic plasma membrane; furthermore, their dendrites presented with distal swellings. The neuron in Figure 1K had a hairy soma with numerous fine sprouts. Figure 1L shows a neuron with shrunken soma and dendrites withered to short thin processes. However, its axon descends giving rise to oblique ascending collaterals which indicated that this dying cell is a pyramidal neuron.

**Figure 2:**
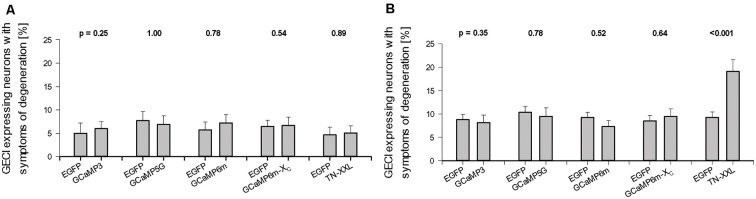
Effect of prolonged genetically encoded calcium indicator (GECI) expression on neuronal degeneration. **(A)** DIV 14. **(B)** DIV 22–25. Percentages of GECI expressing neurons (mean ± SEM) displaying symptoms of degeneration after 10 and >16 days of overexpression plotted against the batch-internal controls. Percentages vary from culture to culture, but none of the GECIs presented with higher a readout at DIV 14. Same at DIV 22–25, except for TN-XXL transfectants which in all batches assessed displayed more transfectants with dendritic beading, shrunken somata, withering dendrites, or an entirely dissolved structure. In every culture, only the completely labeled neurons were scored as “healthy” or as displaying symptoms; in sum 100%. The numbers of assessed Organotypic cultures (OTCs) per condition and the number of culture batches are given in Supplementary Table S1. The *p* values determined with the Mann–Whitney rank-sum test are given above each set of bars.

These symptoms, either alone or in combination, occur in transfected neurons of all conditions, control, and GECI expression. Considered in the following assessment was the fraction of completely stained neurons of every OTC, and those, in turn, are a subset of all neuronal transfectants many of which express at levels so low that distal dendrites can not be safely assessed. For obvious reasons such weakly stained neurons were not assessed for degeneration or morphometry. On average, at DIV 14 the proportion of transfectants with symptoms was about 5% (Figure 2A). Proportions of neurons with degeneration varied slightly from batch to batch, but there had been no statistically significant difference with either one of the GECIs at DIV 14 (Figure 2A). GCaMP6m expression has been reported to display a tripling of apoptosis with annexin-V staining after 10 days in dissociated culture (Yang et al., 2018). At DIV 22–25, the proportion of neurons with symptoms was between 5 and 10% for the GCaMP variants; again, there was no statistical difference to the batch-internal controls. However, a substantially higher proportion of TN-XXL transfectants displayed symptoms of degeneration at DIV 25 suggesting that toxicity had increased with time under TN-XXL (Figure 2B). The numbers of assessed OTCs per condition and the number of culture batches are given in Supplementary Table S1.

**Figure 3:**
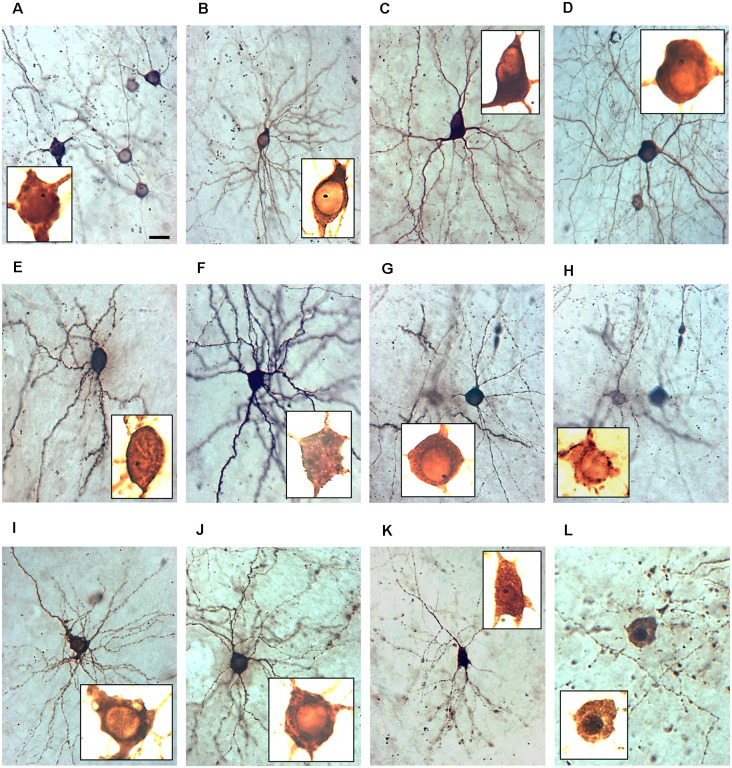
The nuclear phenotype of GECI transfectants. All micrographs have been taken at 400× magnification at DIV 22–25. The insets show the somata photographed at 1,000× magnification with extreme overexposure to visualize the inner structure and nuclei, all scaled identically. Note that nuclei often harbored a single gold particle. The dark staining of the nucleus is an indicator of the filling with calcium indicator protein. **(A)** Group of five GCaMP6m expressing neurons, one with an occluded nucleus stained as dark as the cytosol. **(B)** GCaMP5G expressing healthy pyramidal neurons with the translucent nucleus. **(C)** GCaMP3 expressing healthy interneuron with the translucent nucleus. **(D)** GCaMP5G expressing healthy interneuron with the translucent nucleus. **(E)** TN-XXL expressing healthy pyramidal neurons with the occluded nucleus. **(F)** GCaMP3 expressing healthy pyramidal neurons with the occluded nucleus. **(G)** TN-XXL expressing healthy interneuron with the translucent nucleus. **(H)** TN-XXL expressing pyramidal cells with a leaky soma and a translucent nucleus surrounded by immunoreactive aggregates. **(I)** GCaMP6m-X_C_ expressing pyramidal cell with vacuolar inclusions and translucent nucleus. **(J)** TN-XXL expressing degenerating pyramidal cells with swellings along a dendrite and translucent nucleus. **(K)** TN-XXL expressing degenerating pyramidal cell with swellings along all dendrites and occluded nucleus. **(L)** TN-XXL expressing dead neurons with a dark nucleus surrounded by a cloud of former dendritic swellings and a dark nucleus. Pial surface to the top. Scale bar in **(A)**: 20 μm for all 400× micrographs. The 1,000× micrographs are all cropped to show the soma and all are scaled identically to allow comparison of nuclear size.

The accumulation of GECIs in the nucleus has been discussed as a major cause of cellular impairments. Yang et al. (2018) show that the majority of neurons have nuclear fills at 2 weeks under GECI (GCaMP3, GCaMP6m). We assessed neurons expressing the GECIs at weak to moderate intensity, which had the nucleus filled or occluded such that it was no longer discernible as a large translucent organelle, but rather had a staining intensity indiscernible from the surrounding cytosol or being even stronger labeled than the surrounding cytosol. We assessed the dark staining of the nucleus in a subset of GECI expressing cells as nuclear filling with calcium indicator protein. Representative examples are shown in Figure 3. For all GECIs we found morphologically healthy neurons with clear (Figures 3A–D,G,H) and with occluded nuclei (Figures 3E,F). Occasionally, the nucleus was still transparent but surrounded by clumps of immunoreactive material, presumably GECI protein stuck at the endoplasmatic reticulum (Figure 3H). Vice versa, we found neurons with symptoms of degeneration having clear (Figures 3I,J) nuclei as well as having occluded nuclei (Figures 3K,L). The occluded nuclei (Figures 3K,L) were often pyknotic compared to the translucent nuclei.

**Figure 4:**
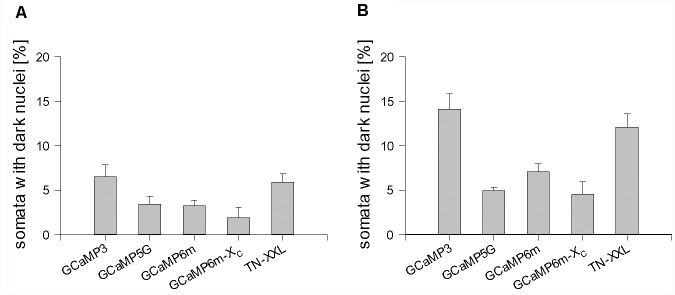
Percentage of GECI expressing neuronal somata with the dark, occluded nucleus. **(A)** DIV 14. **(B)** DIV 22–25. The average per OTC was plotted as mean ± SEM. The total number of assessed somata, OTC, and independent preparations are reported in Supplementary Table S1.

Counting the proportions revealed that all GECI expressing neuronal populations had a few somata with the occluded nucleus at DIV 14 (Figure 4A). The proportions nearly doubled until DIV 22–25 (Figure 4B). GCaMP3 and TN-XXL transfectants displayed the highest percentage of neurons with occluded nuclei differing significantly from the other conditions. When selectively assessing the TN-XXL-expressing neurons with mild or severe dendritic beading, of 61 neurons 41 had an occluded nucleus (67%) suggesting that nuclear filling increases along the final path to disintegration. GCaMP5G, GCaMP6m, and GCaMP6m-X_C_ transfectants had moderate proportions of dark nuclei (Figure 4). EGFP filled the nuclei of many cells from the beginning of expression. Accordingly, of the EGFP control transfectants about 80% had nuclei often darker than the cytosol (see Figure 1), however, that seemingly did not affect morphological maturation and cellular health.

To confirm the nuclear filling, confocal imaging was done exemplarily with GCaMP6m transfectants. Line scans revealed that GCaMP6m was excluded from the nucleus of healthy neurons (Supplementary Figure S1A); intensities alternated with DAPI and chromocenters gave distinct peaks. In nuclear-filled cells, the nucleus often had almost the same GCaMP6m fluorescence intensity than the cytosol, and DAPI no longer revealed the sharp peaks of chromocenters (Supplementary Figure S1B), suggesting a nuclear impairment. It has been reported that nuclear filling alters the physiology (Yang et al., 2018). In OTC we found that neurons with translucent nuclei display appreciable levels of spontaneous activity with calcium events of moderate to high amplitude (Supplementary Figures S1C,D). In contrast, neurons with filled nuclei might display similar patterns of calcium events at least part of the time, but many displayed aberrant calcium events of large width or very small amplitudes (Supplementary Figures S1E,F). Other cells were silent lacking calcium events during the recording period. By experience and as a qualitative criterion, neurons with permanent high nuclear fluorescence tended to be rather silent. This led to the decision to reconstruct only GECI expressing neurons that displayed a nucleus brighter and more translucent than the surrounding cytosol and to not sample neurons with dark, occluded, pyknotic nuclei, even when their dendrites were still looking healthy.

### Impairment of Pyramidal Cell Dendritic Growth

**Figure 5:**
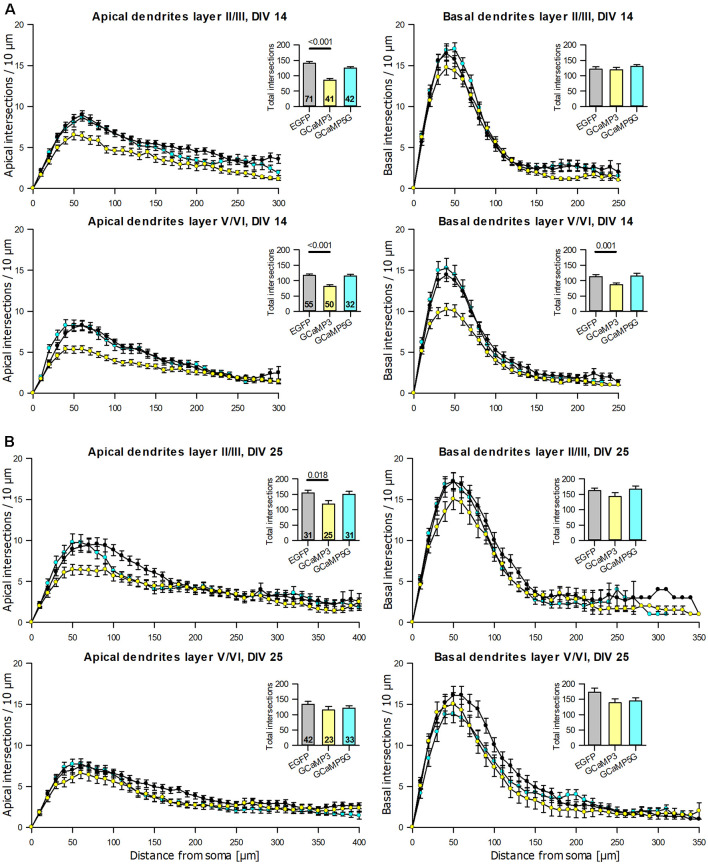
Sholl analyses of apical and basal dendrites of pyramidal neurons of layers II/III and V/VI expressing GCaMP3 and GCaMP5G, respectively, vs. batch-internal EGFP expressing neurons. **(A)** DIV 14. **(B)** DIV 25. Insets report total intersections. *P* values of the Mann–Whitney rank-sum test vs. control performed after the ANOVA on ranks indicated a significant difference vs. control.

The anti-EGFP antibody staining came at variable intensities suggestive of varying expression levels. For the reconstruction, the intensely labeled cells like those shown above (see Figures 1B,E,F) were preferred, but nearly all adequately labeled neurons with translucent nuclei per culture had been reconstructed by the Neurolucida-competent authors who were blinded to condition; there was no preselection of which cell should be reconstructed. All reconstructions were printed to scale and cross-checked with a Camera lucida at 400× magnification for correctness and classification of type by the senior author who was also blinded to conditions. Pyramidal neurons of supra- and infragranular layers expressing GCaMP3 were consistently underdeveloped at DIV 14 with apical and basal dendrites being shorter and/or less branched (Table 2A). The hypomorphy persisted at DIV 25 (Table 2B). GCaMP5G expressing pyramidal cells were not impaired at DIV 14 (Table 2A), but smaller and less branched trees occurred in infragranular pyramidal cells at DIV 25 (Table 2B). The Sholl analysis (Figure 5A) revealed that apical dendrites of GCaMP3 transfectants of both layers were substantially less branched over the entire length of the tree (10 μm bins). Also, the total intersections were significantly less than in control neurons at all distances (insets in the graphs). GCaMP5G expressing neurons, by contrast, were not different from control. At DIV 25, the dendritic complexity of GCaMP3 expressing neurons was below control (Figure 5B). Also, the GCaMP5G expressing neurons of infragranular layers had a reduced dendritic branching (Figure 5B).

**Table 2:** Measures of apical and basal dendrites of pyramidal neurons of layers II/III and V/VI expressing GCaMP3 and GCaMP5G, respectively, vs. batch-internal EGFP expressing neurons as control.

Pyramidal cells overexpressing GCaMP3 and GCaMP5G at DIV 14 and DIV 25				
	Pyramidal cells in layers II/III		Pyramidal cells in layers V/VI	
	ADL (*n*)	BDL	ADL (*n*)	BDL
Age, condition (Number of batches)	Segments	Segments	Segments	Segments
**(A) DIV 14** Control for GCaMP3 and GCaMP5G (4)	1716 ± 74 (71)	336 ± 15	1468 ± 61 (55)	308 ± 19
	37.1 ± 1.8	8.9 ± 0.5	31.7 ± 1.8	7.8 ± 0.4
GCaMP3 (4)	**1072 ± 77** (41)	320 ± 24	**1037 ± 69** (50)	300 ± 20
	**22.9 ± 1.5**	7.5 ± 0.5	**21.8 ± 1.9**	**6.7 ± 0.5**
GCaMP5G (3)	1603 ± 69 (42)	315 ± 18	1470 ± 71 (32)	326 ± 21
	36.1 ± 1.7	7.7 ± 0.5	32.8 ± 1.9	8.0 ± 0.5
*ANOVA on ranks vs. control*	*<0.001*	*0.61*	*<0.001*	*0.47*
	*<0.001*	*0.094*	*<0.001*	*0.022*
*Mann–Whitney test GCaMP3 vs. control*	*<0.001*		*<0.001*
	*<0.001*		*<0.001*	*0.022*
**(B) DIV 25** Control for GCaMP3 and GCaMP5G (4)	1980 ± 125 (31)	439 ± 28	1876 ± 108 (38)	446 ± 33
	34.9 ± 2.1	8.7 ± 0.5	29.3 ± 1.4	8.3 ± 0.6
GCaMP3 (4)	**1449 ± 125** (25)	374 ± 27	**1432 ± 140** (23)	**337 ± 31**
	**24.6 ± 2.0**	7.3 ± 0.6	24.6 ± 2.3	7.1 ± 0.6
GCaMP5G (3)	1901 ± 126 (31)	394 ± 23	**1481 ± 99** (32)	403 ± 42
	32.0 ± 2.0	7.6 ± 0.4	26.7 ± 2.1	7.6 ± 0.6
*ANOVA on ranks vs. control*	*0.015*	*0.417*	*0.009*	*0.003*
	*<0.001*	*0.082*	*0.096*	*0.040*
*Mann–Whitney test*	*0.008*		*0.025*	*0.012*
*GCaMP3 vs. control*	*<0.001*		
*Mann–Whitney test*			*0.005*
*GCaMP5G vs. control*		

*(A) DIV 14. (B) DIV 25. Given are the mean ± SEM, and n, the number of cells analyzed. P values of the Mann–Whitney rank-sum test vs. control performed after the ANOVA on ranks are in italics. ADL, apical dendritic length (μm); BDL, mean (per cell) basal dendritic length (μm); segments, number of dendritic segments. Significant values are bold and *p*-values are shown in italic*.

**Figure 6:**
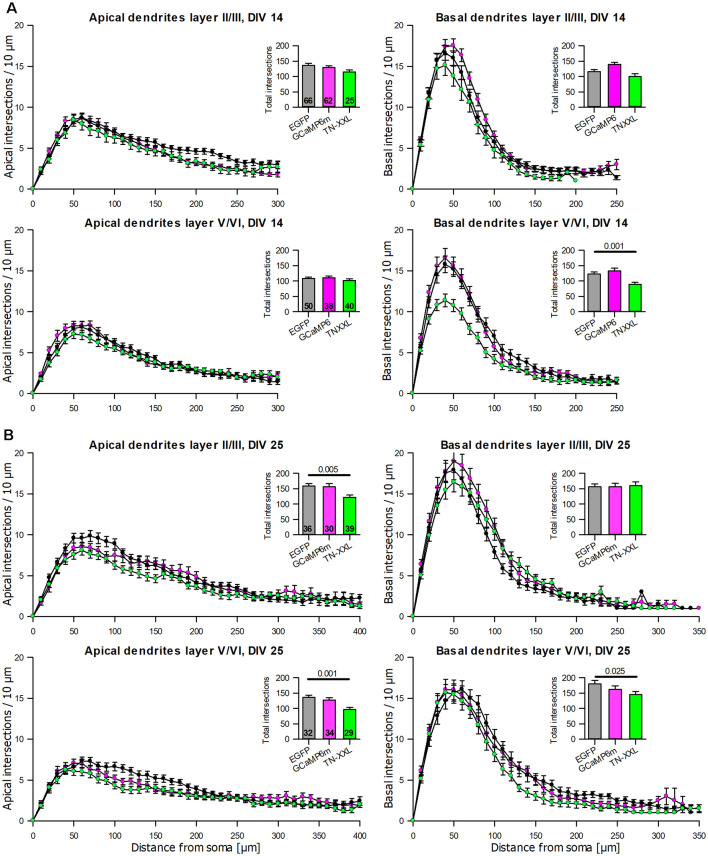
Sholl analyses of apical and basal dendrites of pyramidal neurons of layers II/III and V/VI expressing GCaMP6m and TN-XXL, respectively, vs. batch-internal EGFP expressing neurons. **(A)** DIV 14. **(B)** DIV 25. Insets report total intersections. *P* values of the Mann–Whitney rank-sum test vs. control performed after the ANOVA on ranks indicated a significant difference vs. control.

The dendritic complexity of the GCaMP6m expressing pyramidal neurons was within the variation of the batch-internal control neurons at DIV 14 and DIV 25 (Tables s 3A,B). TN-XXL transfectants had a trend towards shorter and less branched basal dendrites at DIV 14 (Table 3A). The dendritic complexity of TN-XXL expressing pyramidal neurons was significantly impaired in both laminar compartments at DIV 25 (Table 3B). The Sholl analysis revealed first deficits in infragranular cell basal branching at DIV 14 (Figure 6A), and a massive deficit in apical and partly basal dendritic arborization at DIV 25; also, mean total intersections were lower (Figure 6B).

**Table 3:** Measures of apical and basal dendrites of pyramidal neurons of layers II/III and V/VI expressing GCaMP6m and TN-XXL, respectively, vs. batch-internal EGFP expressing neurons as control.

Pyramidal cells overexpressing GCaMP6m and TN-XXL at DIV 14 and DIV 25				
	Pyramidal cells in layers II/III		Pyramidal cells in layers V/VI	
	ADL (*n*)	BDL	ADL (*n*)	BDL
Age, condition (Number of batches)	Segments	Segments	Segments	Segments
**(A) DIV 14** Control for GCaMP6m and TN-XXL (5)	1729 ± 71 (66)	340 ± 18	1387 ± 73 (48)	347 ± 22
	37.5 ± 1.9	8.5 ± 0.4	29.4 ± 2.0	8.7 ± 0.5
GCaMP6m (5)	1662 ± 69 (62)	332 ± 18	1432 ± 90 (38)	349 ± 24
	35.1 ± 1.7	8.0 ± 0.4	30.0 ± 2.1	8.1 ± 0.6
TN-XXL (3)	1498 ± 102 (25)	340 ± 33	1312 ± 63 (40)	**267 ± 18**
	33.9 ± 2.3	9.0 ± 0.8	28.2 ± 1.8	**6.5 ± 0.4**
*ANOVA on ranks vs. control*	*0.196*	*0.944*	*0.764*	*0.004*
	*0.715*	*0.562*	*0.833*	*0.055*
*Mann–Whitney test*				*0.003*
*TN-XXL vs. control*				*0.048*
**(B) DIV 25** Control for GCaMP6m and TN-XXL (4)	1951 ± 118 (36)	391 ± 23	1783 ± 128 (32)	492 ± 37
	35.3 ± 1.8	8.4 ± 0.6	27.6 ± 1.9	9.6 ± 0.9
GCaMP6m (3)	2003 ± 130 (30)	438 ± 25	1682 ± 102 (34)	395 ± 24
	33.4 ± 2.3	8.9 ± 0.5	28.5 ± 2.1	8.1 ± 0.6
TN-XXL (4)	**1522 ± 91** (39)	451 ± 33	**1265 ± 89** (27)	399 ± 25
	**27.4 ± 1.5**	9.0 ± 0.6	**22.6 ± 1.9**	8.0 ± 0.6
*ANOVA on ranks vs. control*	*0.007*	*0.242*	*0.008*	*0.084*
	*0.034*	*0.692*	*0.085*	*0.395*
*Mann–Whitney test*	*0.018*		*0.011*
*TN-XXL vs. control*	*0.004*		

*(A) DIV 14. (B) DIV 25. Given are the mean ± SEM, and n, the number of cells analyzed. P values of the Mann–Whitney rank-sum test vs. control performed after the ANOVA on ranks are in italics. ADL, apical dendritic length (μm); BDL, mean (per cell) basal dendritic length (μm); segments, number of dendritic segments. Significant values are bold and *p*-values are shown in italic*.

**Figure 7:**
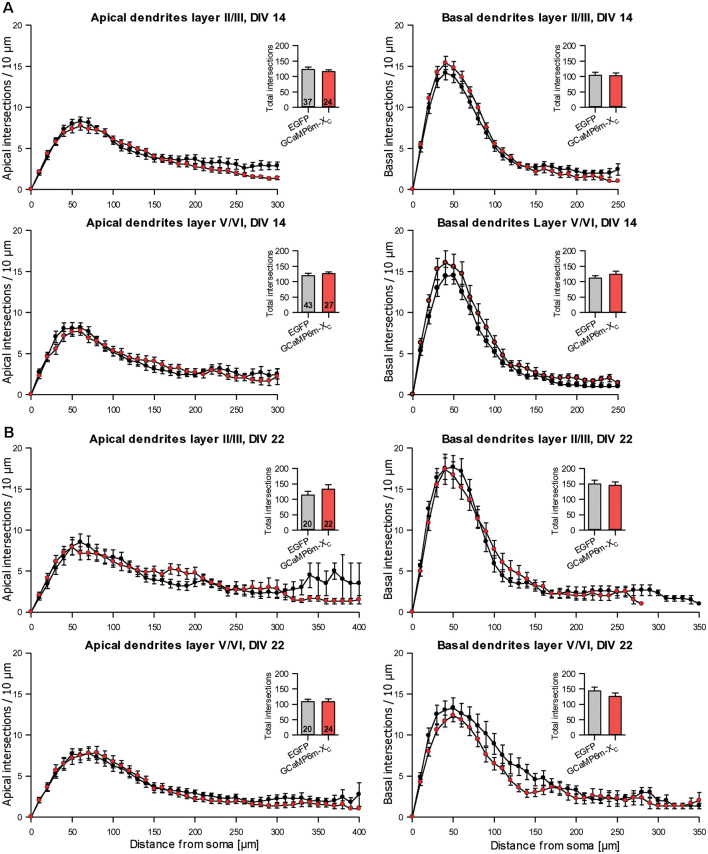
Sholl analyses of apical and basal dendrites of pyramidal neurons of layers II/III and V/VI expressing GCaMP6m-X_C_ vs. batch-internal EGFP expressing neurons. **(A)** DIV 14. **(B)** DIV 22. Insets report total intersections.

During the analysis, GCaMP6m-X_C_ was published (Yang et al., 2018) and reported to cause less damage due to the shielded CAM domain. Similar to GCaMP6m, the length and branching of pyramidal neurons expressing GCaMP6m-X_C_ from DIV 4–14 or DIV 4–22 were not different from batch-internal control (Tables s 4A,B). Also, the Sholl analysis revealed that the branching largely follows that of control cells (Figure 7).

**Table 4:** Measures of apical and basal dendrites of pyramidal neurons of layers II/III and V/VI expressing GCaMP6m-X_C_ vs. batch-internal EGFP expressing neurons as control.

Pyramidal cells overexpressing GCaMP6m-X_C_ at DIV 14 and DIV 22				
	Pyramidal cells in layers II/III		Pyramidal cells in layers V/VI	
	ADL (*n*)	BDL	ADL (*n*)	BDL
Age, condition (Number of batches)	Segments	Segments	Segments	Segments
**(A) DIV 14** Control for GCaMP6m-X_C_ (4)	1653 ± 110 (37)	332 ± 20	1333 ± 103 (24)	341 ± 22
	34.4 ± 1.9	8.3 ± 0.5	29.8 ± 2.4	8.8 ± 0.6
GCaMP6m-X_C_ (4)	1526 ± 85 (43)	360 ± 18	1358 ± 113 (27)	324 ± 22
	32.1 ± 1.9	8.5 ± 0.5	31.4 ± 2.9	8.3 ± 0.7
*Mann–Whitney test vs. control*	*0.265*	*0.240*	*0.992*	*0.670*
	*0.154*	*0.891*	*0.835*	*0.313*
**(B) DIV 22** Control for GCaMP6m-X_C_ (3)	1625 ± 154 (18)	399 ± 27	1590 ± 103 (22)	365 ± 29
	31.4 ± 3.3	9.6 ± 0.4	28.6 ± 1.8	7.0 ± 0.4
GCaMP6m-X_C_ (3)	1675 ± 133 (21)	362 ± 20	1479 ± 116 (25)	378 ± 24
	30.6 ± 2.5	8.3 ± 0.7	28.0 ± 3.2	7.3 ± 0.4
*Mann–Whitney test vs. control*	*0.833*	*0.217*	*0.409*	*0.426*
	*0.989*	*0.030*	*0.422*	*0.573*

*(A) DIV 14. (B) DIV 22. Given are the mean ± SEM, and n, the number of cells analyzed. P values of the Mann–Whitney rank-sum test vs. control are in italics. ADL, apical dendritic length (μm); BDL, mean (per cell) basal dendritic length (μm); segments, number of dendritic segments. Significant values are bold and *p*-values are shown in italic*.

Finally, the soma area of the reconstructed pyramidal neurons was determined as a proxy for the size of the cell bodies. There were no differences between the soma size of GECI expressing pyramidal neurons to EGFP expressing control neurons (Supplementary Figures S2A,B). The impairment of dendritic elongation and branching of GCaMP3, GCaMP5G, and TN-XXL thus occurred in the absence of measurable deficits in soma size presumably because only the healthy-looking neurons had been reconstructed.

### Little Effects on Multipolar Interneurons

Non-pyramidal multipolar neurons with smooth or sparsely spinous dendrites reconstructed from the same set of cultures were by and large unaffected. Only GCaMP3 expressing interneurons presented with significantly shorter and less branched dendrites and smaller somata at DIV 14 when compared to batch-internal control cells. Branching was not different from control (Table 5A). This seemed to be a transient impairment of differentiation because GCaMP3 expressing interneurons at DIV 25 no longer differed from control. The other GECIs did not impair interneuronal differentiation (Tables s 5B,C; Supplementary Figure S2). The number of primary dendrites was not altered suggesting that GECI expression does not impair neuritogenesis.

**Table 5:** Measures of dendrites of multipolar interneurons.

Interneurons overexpressing GECIs at DIV 14, DIV 22, and DIV 25						
	MDL (*n*)	PD	Soma size	MDL (*n*)	PD	Soma size
Condition (Number of batches)	MDS			MDS		
	DIV 14			DIV 25		
**(A)** Control for GCaMP3 and GCaMP5G (5; 4)	468 ± 38 (34)	5.4 ± 0.4	254 ± 123	563 ± 41 (48)	5.3 ± 0.3	273 ± 90
	7.1 ± 0.5			7.1 ± 0.5
GCaMP3 (5; 4)	**359 ± 30** (29)	5.0 ± 0.3	**179 ± 57**	569 ± 36 (31)	5.1 ± 0.3	289 ± 90
	5.6 ± 0.5			7.4 ± 0.6
GCaMP5G (4; 3)	427 ± 27 (27)	5.1 ± 0.2	220 ± 59	526 ± 70 (24)	5.8 ± 0.3	310 ± 105
	6.4 ± 0.5			6.2 ± 0.7
*ANOVA on ranks vs. control*	*0.040*	*0.855*	*0.002*	*0.292*	*0.343*	*0.324*
	*0.167*			*0.110*		
*Mann–Whitney test*	*0.022*		*0.001*			
*GCaMP3 vs. control*						
**(B)** Control for GCaMP6m and TN-XXL (5; 6)	498 ± 37 (31)	4.5 ± 0.2	253 ± 123	542 ± 27 (49)	5.3 ± 0.3	258 ± 70
	7.6 ± 0.6			7.1 ± 0.4
GCaMP6m (5; 3)	429 ± 29 (39)	5.6 ± 0.3	240 ± 72	591 ± 54 (31)	5.5 ± 0.3	*295* ± 82
	7.0 ± 0.4			7.0 ± 0.7
TN-XXL (3; 4)	477 ± 55 (16)	5.0 ± 0.5	195 ± 86	498 ± 51 (27)	5.2 ± 0.3	270 ± 98
	6.9 ± 0.7			6.6 ± 0.6
*ANOVA on ranks vs. control*	*0.285*	*0.070*	*0.158*	*0.216*	*0.864*	*0.137*
	*0.776*			*0.643*		
	**DIV 14**			**DIV 22**		

**(C)** Control for GCaMP6m-X_C_ (4; 3)	452 ± 43 (22)	5.0 ± 0.4	225 ± 43	583 ± 63 (31)	5.4 ± 0.3	226 ± 68
	7.5 ± 0.8			7.8 ± 0.7
GCaMP6m-X_C_ (4; 3)	477 ± 65 (17)	5.4 ± 0.4	261 ± 62	628 ± 52 (21)	4.9 ± 0.3	256 ± 80
	7.3 ± 0.8			7.6 ± 0.7
*Mann–Whitney test vs. control*	*0.966*	*0.318*	*0.067*	*0.225*	*0.143*	*0.112*
	*0.864*			*0.978*		

*(A) GCaMP3 and GCaMP5G. (B) GCaMP6m and TN-XXL. (C) GCaMP6m-X_C_, all vs. batch-internal EGFP expressing neurons as control at DIV 14 (left) and DIV 22 or 25, respectively (right). Given are the mean ± SEM, and n, the number of cells analyzed. MDL, mean (per cell) dendritic length (μm); MDS, mean (per cell) basal dendritic segments; PD, number of primary dendrites. The Soma area is given in μm^2^. P values of a one-way ANOVA on ranks or a Mann–Whitney test are shown in italics. Significant values are bold and p-values are shown in italic*.

### GCaMP3 Effects on Differentiated Neurons

The substantial impairment evoked by early postnatal expression of GCaMP3 let us test if dendritic deficits could also be evoked when transfecting nearly differentiated neurons at DIV 20 followed by expression until DIV 30. Analyzing a set of cells sampled from three batches we found that dendritic dimensions of DIV 30 neurons were by and large in the range of DIV 25 neurons indicating that maturation had reached a plateau. GCaMP3 transfected pyramidal neurons and interneurons had dendritic complexities not significantly different from batch-internal EGFP control transfectants (Supplementary Table S2) with similar soma sizes (mean ± SEM 182 ± 7.1 μm^2^ vs. 194 ± 5.1 μm^2^ in control). Neurons with symptoms of degeneration (mean ± SEM 11.5 ± 3.1%) were similar to EGFP control neurons (8.7 ± 1.2%), and the percentage of neurons with occluded nuclei (10.9 ± 1.3%) was similar to the percentage determined at DIV 25 (Figure 2; see Supplementary Table S1 for the numbers). Together, this suggested that a late expression for 10 days at least does not evoke neuronal deficits.

### Fe65 Nuclear Spheres Alter Dendritic Growth

The GECI results suggest that cytosolic impairment reduces dendritic growth. However, a nuclear impairment may also contribute. To this end, we have looked at a protein that shares one aspect with the GECIs—nuclear localization and nuclear impairment. The rationale is as follows. Fe65 is an adaptor protein extensively analyzed in the context of Alzheimer’s disease because it binds to the amyloid precursor protein (APP) intracellular domain (McLoughlin and Miller, 2008). Fe65 is predominantly expressed in neurons with high expression hippocampus, but also in the neocortex, and found in excitatory synapses and neurite growth cones (Masin et al., 2006). Developmentally, Fe65 expression starts around embryonic day 15 in mouse, is low perinatally, and increases after postnatal day 10 (Kesavapany et al., 2002). Fe65 may be involved in regulating axonal branching (Ikin et al., 2007), and a knockout leads to mismigration and axonal growth impairments *via* modulation of the actin cytoskeleton (McLoughlin and Miller, 2008). Functionally, a lack of Fe65 leads to defects in hippocampal spine plasticity and long-term potentiation (Strecker et al., 2016). Fe65 may have a role in intracellular calcium homeostasis because a knockout of Fe65 evokes in hippocampal neurons an upregulation of Serca2 (Nensa et al., 2014), which is known to concur with ER stress and unfolded protein response (Højmann Larsen et al., 2001). A prominent feature is that Fe65 enters the nucleus forming nuclear spheres (Schrötter et al., 2013; Nensa et al., 2014). These spheres are found in neuronal nuclei of postmortem human brain from Alzheimer’s disease patients and are discussed as leading to a transcriptional alteration and cell cycle re-entry followed by neuronal degeneration (Kolbe et al., 2016; Bukhari et al., 2017). Together, this suggested that Fe65 overexpression could be a model where disruption of nuclear function may lead to dendritic deficits. Indeed, mouse models for Alzheimer’s disease-related mutations have revealed that the complexity of apical and in particular basal pyramidal cell dendrites in the cortex and hippocampus become substantially reduced (Alpár et al., 2006; Šišková et al., 2014; Mehder et al., 2020). Similarly, in the cortex of senile aged humans dendrites of supragranular pyramidal cells display changes of the cell body, dendritic swellings, and a loss of basal dendrites followed by apical deficits and cell death (Scheibel et al., 1975; Baloyannis, 2009). This led us to test if Fe65 forms the potentially toxic nuclear spheres in early postnatal cortical neurons, and if Fe65 overexpression influences dendritic growth. Since the nuclear localization of Fe65 can be a trigger for apoptosis in particular when co-expressed with the interacting proteins such as the histone acetyltransferase TIP60 (Matz et al., 2015), we decided to express Fe65 alone at the time when it naturally increases in the brain (Kesavapany et al., 2002). We analyzed the neurons within the time window selected for GECI expression, but already after about 50 h of Fe65 overexpression to not sample potentially degenerating neurons.

Transfection with EGFP-tagged Fe65 together with mCherry at DIV 12 yielded within 12–24 h after transfection strongly fluorescent nuclear aggregates (Supplementary Figure S3A) as previously reported for HEK cells (Schrötter et al., 2013; Nensa et al., 2014) and neurons in postmortem human brain (Kolbe et al., 2016). Compared to mCherry-stained control cells the Fe65 expressing neurons did not show symptoms of degeneration at DIV 14 (Supplementary Figure S3B). Reconstruction of transfectants at DIV 14/15 revealed that as little as 50 h of FE65 overexpression had a negative impact on basal but not apical dendritic growth of pyramidal cells of supragranular layers (Supplementary Figure S3C; Supplementary Table S3) which is also evident in the Sholl analysis (Supplementary Figure S3D). Basal dendrites remained almost 20% shorter than the average of the control and were significantly less branched with a reduction of total intersections in the Sholl analysis. For comparison, the GCaMP3-expressing neurons at DIV 14 often had ≥30% deficits in length and branching. Ontogenetically older infragranular pyramidal cells and interneurons were not affected (Supplementary Table S3). This suggested that a nuclear impairment can have a fast negative influence on arborization and supported the view that neurons at this developmental period are highly susceptible.

## Discussion

Calcium indicators have been finally confirmed as being calcium buffers (McMahon and Jackson, 2018). Our study looks at the variable “time under GECI” within critical time windows for dendrite growth, the fast growth phase until the end of the second week, and a slower growth phase with remodeling towards 1 month postnatal (Hamad et al., 2011, 2014; Ramaswamy and Markram, 2015; Jack et al., 2018). Our data suggest that some GECIs are more of a concern than others. GCaMP3 and TN-XXL, and to some extend GCaMP5G caused more structural damage after prolonged-expression than the other two GECIs tested. In other words, our results suggested that GECIs with the highest K_D_ (Rose et al., 2014) are more detrimental than those with lower calcium-binding affinity. Some of the effects we showed might appear numerically small. However, we reconstructed the healthy-looking neurons with translucent nuclei, omitting those with occluded nuclei and those with symptoms of degeneration such as withering dendrites. This suggested that subtle to severe growth delays occur and persist over time under GECI.

The growth impairment seemed not to be evoked solely by nuclear filling. First, only neurons with translucent nuclei were reconstructed in our sample. Second, the impairment with GCaMP3 already occurred at DIV 14 when rates of nuclear filling were comparably low in all GECI expressing populations. Third, neurons expressing TN-XXL, the GECI with the highest K_D_ value, tended to be hypomorphic already at DIV14 and were significantly hypomorphic at DIV 25 without particularly high rates of nuclear filling. However, the rate of degeneration observed at DIV 25 with TN-XXL coincided with a higher rate of nuclear filling in degenerating neurons. Thus, detrimental effects on dendritic differentiation presumably occurred by events that had happened already at the level of the neuron’s cytosol. GCaMP3, GCaMP5G and GCaMP6m nuclear-filled neurons in dissociated cultures display shorter neurites and smaller amplitudes of calcium events (Yang et al., 2018), and our imaging results confirmed this. GCaMP3 has been reported to impair transcription in response to membrane excitation such that the phospho-CREB phosphorylation is lower in GCaMP3-nuclear filled neurons (Yang et al., 2018). Results obtained from the overexpression of foreign proteins always need to be carefully interpreted. For instance, microbial opsins became indispensable tools, but it has been reported that the proteins do not always enrich in the plasma membrane. Rather, they may accumulate in the cytosol or at the endoplasmatic reticulum leading to ER stress *via* an unfolded protein response which can be toxic (Garita-Hernandez et al., 2018). Genetically encoded fluorescent periplasmatic glutamate sensor proteins faithfully report synaptic vesicle release, but alter glutamate diffusion, activation of extrasynaptic receptors, and uptake into astrocytes (Armbruster et al., 2019).

GCAMP3 and TN-XXL impaired apical and basal dendritic growth. Earlier work showed that apical dendrites are more malleable by alterations of electrical activity than basal dendrites. For instance, overexpression of calcium-permeable and calcium-impermeable flip-spliced (longer channel open times) AMPA receptors as well as the trafficking-competent transmembrane AMPA receptor regulatory proteins evoke an elongation and branching of apical dendrites driven by an increase in the amplitude of calcium events (Hamad et al., 2011, 2014). Further, kainate-evoked network activity as well as overexpression of GluK2 evoke an elongation and branching of apical dendrites, presumably driven by the increased frequency of calcium events (Jack et al., 2018). Basal dendrites have not been altered in these experiments which suggested that the two dendritic compartments employ different growth mechanisms. Now, basal dendrites are affected by GCaMP3 and TN-XXL. In hippocampal CA1 pyramidal neurons, basal dendritic complexity *in vivo* as well as total dendritic complexity *in vitro* is reduced after introducing the cytosolic calcium buffer protein parvalbumin to the nucleus. Mechanistically, this leads to enhanced nuclear calcium buffering, the subsequently lower expression of the dendrite maintenance factor vascular endothelial growth factor D, which compromises dendritic complexity as well as cognitive abilities (Mauceri et al., 2011, 2015; Hemstedt et al., 2017). Thus, nuclear impairment can not be excluded.

Indeed, a rather short overexpression of Fe65 evoked a delay in the basal dendritic growth of supragranular pyramidal neurons. Our results seem to be at odds with the neurite-growth promoting action of Fe65 (Sabo et al., 2003; Cheung et al., 2014; Li et al., 2018). However, these studies assessed neurite growth in embryonic dissociated neurons at even more immature stages and focussed on the longest neurite which is usually the nascent axon the growth of which is influenced by Fe65 (McLoughlin and Miller, 2008). Our results now show a specific defect of dendritic growth in more differentiates neurons growing in their normal 3D environment. Supragranular neurons have been reported to be particularly vulnerable to dendritic pathology in Alzheimer’s disease (Scheibel et al., 1975; Alpár et al., 2006; Baloyannis, 2009; Stranahan and Mattson, 2010). A substantial reduction of basal dendritic branching has been shown by Sholl analyses of Golgi-impregnated pyramidal neurons of children and adults with Down syndrome which is associated with AD-like dementia (Takashima et al., 1994). Together, this supports the view that nuclear alterations can negatively influence dendritic growth.

Hypomorphic dendrites have been shown to display an altered reaction-diffusion time for signal transduction and calcium propagation, and this can lead to an enhanced somatodendritic coupling caused by misplaced or locally higher amounts of ion channels; modeling shows that hypomorphic neurons may generate more spikes for smaller inputs (Šišková et al., 2014; Dhupia et al., 2015). Neurons overexpressing the ASD-related protein ubiquitin-protein ligase Ube3A/E6AP excessively prune branches and present with fewer and less branched dendrites which display fewer protrusions and changes in a synaptic activity such as reduced mEPSC frequency (Smith et al., 2011; Khatri et al., 2018). This suggested that underdeveloped neurons are not properly integrated into the circuitry.

GCaMP3 has been widely used in transgenic studies with expression during neuronal maturation. However, morphological defects, should they have occurred, would have gone unnoticed because GCaMP3 has not been the variable under investigation. The DIV 20–30 data suggested that overexpression in adult neurons is less detrimental. Further, the troponin moiety of TN-XXL is believed to not interact with neuronal molecules (Heim and Griesbeck, 2004), however, some aberrant behavior has been reported in TN-XXL transgenic mice (Direnberger et al., 2012). GCaMP6m and its derivate GCaMP6m-X_C_ behaved quite “neuron-friendly” in our study. However, neonatal viral transfection of cochlear neurons with GCaMP6 followed by a 3-week expression has been reported to impair vesicle release and short-term depression at the calyx of Held synapse, albeit without affecting quantal events during spontaneous activity (Singh et al., 2018). Transgenic mouse lines expressing CaMK2α-driven GCaMP6 in cortical pyramidal cells have been reported to display episodes of epileptiform activity suggesting that the amount of GCaMP6 produced by the cells at early stages of development is an important culprit: yet, in between these episodes, some aspects of visual cortical processing have remained intact (Steinmetz et al., 2017).

Transgenic approaches with GCaMPs usually target cell types using type-specific promoters, for instance, the neuronal Thy-1 promoter, or the CaMK2 promoter yielding moderate expression levels. To study early developmental events we used CMV promoter-driven expression in young neurons to deliver the CECI protein quickly within a day to pyramidal neurons and interneurons. The foreign protein can accumulate to levels high enough for anti-EGFP antibodies to reveal the full dimension of dendritic trees. The CMV-driven expression alone is unlikely to explain the result because GCAMP6m and GCaMP6m-X_C_ were equally CMV-driven and they did not lead to measurable dendritic alterations. Further, our control cells were labeled by CMV-driven EGFP expression, and we are not aware of reports showing detrimental actions of cytosolic or nuclear EGFP expression. For instance, long-term imaging of GFP expressing neurons in the mouse cortex accessible *via* a cranial window reveals highly stable structures over weeks (Holtmaat et al., 2009) and an intact appearance at the electron microscopic level (Knott et al., 2009). Further, it is unlikely that the detrimental effects had been due to extremely large amounts e.g., of GCaMP3 in the cells. Judged by immunohistochemical staining intensity, the expression levels of the GECIs did vary between cells, and for obvious reasons, only the completely stained cells with healthy appearance were reconstructed. It was not evident that GCaMP3 labeling intensities became much stronger in comparison to e.g., GCaMP6m, and that this had inflicted the impairment of dendritic growth. More likely, the kinetics of GCaMP3 is the culprit. It is reported to have the highest baseline fluorescence suggesting that it binds more calcium and this way causes a stronger dysregulation of calcium homeostasis (Akerboom et al., 2012). Dysregulation in the cytosol and nucleus can impact neuronal function and health (Bading, 2013).

The dendritic architecture of cortical neurons has been correlated with intelligence in humans (Goriounova et al., 2018). The length of the apical dendritic trunk of layer V pyramidal neurons determines excitability and propensity for burst firing (Galloni et al., 2020). So, if cells fail to mature properly with GECI expression in experimental models *in vivo*, the cognitive performance of the individual may be compromised. In conclusion, as recent studies have emphasized (Steinmetz et al., 2017; McMahon and Jackson, 2018; Singh et al., 2018), care is required to interpret results obtained with neurons (and animals) harboring GECI’s for prolonged periods.

## Conclusion

We demonstrated that the overexpression in particular of GCaMP3 and TN-XXL disturbs the morphological differentiation of cortical pyramidal cells and immature interneurons. The morphological development of GCaMP6m and GCaMP6m-X_C_ transfected neurons was not compromised after several weeks of overexpression.

## Data Availability Statement

All datasets presented in this study are included in the article/Supplementary Material.

## Ethics Statement

The animal study and animal protocols were reviewed and approved by Ruhr University Bochum Animal Research Board and the Federal State of North Rhine-Westphalia.

## Author Contributions

AJ and PW designed experiments. IG, AJ, TS, SG, L-MR, and PW performed experiments and data management. IG, AJ, TS, and PW interpreted results. AJ, IG, and PW wrote the manuscript. All authors contributed to the article and approved the submitted version.

## Conflict of Interest

The authors declare that the research was conducted in the absence of any commercial or financial relationships that could be construed as a potential conflict of interest.
