# Supplementary material for: Genetically Encoded Calcium Indicators Can Impair Dendrite Growth of Cortical Neurons
Source: Front Cell Neurosci. 2020 Oct 20;14:570596. doi: 10.3389/fncel.2020.570596 (PMC7606991; doi:10.3389/fncel.2020.570596)

**Supplemental Tables**

**Supplemental Table 1. Analysis of degeneration and occluded nuclei**. Table reports the ages and the constructs tested, the n of neurons assessed, the n of cultures delivering these neurons, and the number of preparations; note that for each batch 4-5 animals are prepared and slices from each individual animal had been allocated to all conditions run with the batch.

|  | **Supplemental Table 1 Analysis of degeneration and occluded nuclei** | | | | | | | |
| --- | --- | --- | --- | --- | --- | --- | --- | --- |
|  | |  | EGFP | GCaMP3 | GCaMP5G | GCaMP6m | TN-XXL | GCaMP6m-X_C_ |
| Degeneration analysis  (neurons/cultures/experiments) | | DIV14 | 2318/61/6 | 1083/29/4 | 799/36/7 | 565/31/4 | 934/25/3 | 262/11/3 |
|  |  | DIV21-25 | 2811/78/6 | 639/37/5 | 506/16/4 | 821/21/4 | 800/25/4 | 905/38/4 |
|  |  | DIV 30 | 153/13/3 | 113/17/3 |  |  |  |  |
| Dark nuclei quantification  (neurons/cultures/experiments)  ( | | DIV 14 | n. d. | 905/26/7 | 798/17/5 | 1203/26/5 | 1262/20/3 | 204/7/2 |
|  |  | DIV21-25 | n. d. | 1497/39/7 | 1596/27/4 | 2056/32/4 | 1945/34/4 | 751/20/4 |
|  |  | DIV 30 | n. d. | 113/17/3 |  |  |  |  |

**Supplemental Table 2. Measures of apical and basal dendrites of pyramidal neurons of layers II/III and V/VI and of interneurons expressing GCaMP3 from DIV 20-30 versus batch-internal EGFP expressing neurons as control**.

**(A)** Pyramidal neurons.

**(B)** Multipolar interneurons.

Given is the mean ± S.E.M., and n, the number of cells analyzed. P values of Mann-Whitney rank sum test versus control are in italics. ADL, apical dendritic length (µm); BDL, mean (per cell) basal dendritic length (µm); segments, number of dendritic segments; MDL, mean dendritic length/cell; MDS, mean dendritic segments/cell; PD, number of primary dendrites.

| **Supplemental Table 2 Pyramidal cells and interneurons overexpressing GECIs at DIV 30** | | | | | | |
| --- | --- | --- | --- | --- | --- | --- |
| A) | Pyramidal cells in layers II/III | | | Pyramidal cells in layers V/VI | | |
| Age, Condition  (Number of batches) | ADL (n)  Segments | BDL  Segments | | ADL (n)  Segments | | BDL  Segments |
| **DIV 30** Control for  GCaMP3 | 1990 ± 116 (21)  29.8 ± 2.8 | 513 ± 42  9.5 ± 0.8 | | 1735 ± 141 (20)  25.7 ± 1.9 | | 495 ± 46  7.8 ± 0.5 |
| GCaMP3 (3) | 1817 ± 174 (17)  29.2 ± 3.6 | 547 ± 36  9.9 ± 0.9 | | 1727 ± 208 (15)  28.6 ± 2.0 | | 385 ± 35  7.5 ± 0.5 |
| *Mann-Whitney test vs control* | *0.265*  *0.648* | *0.282*  *0.834* | | *0.765*  *0.337* | | *0.035*  *0.831* |
|  |  |  | |  | |  |
| B) | Interneurons | | | | | |
|  | MDL (n) | | MDS | | PD | |
| **DIV 30** Control for  GCaMP3 | *601* ± 31 (11) | | *7.8* ± 0.6 | | *5.3* ± 0.5 | |
| GCaMP3 (3) | *595* ± 46 (23) | | *7.6* ± 0.7 | | *4.6* ± 0.3 | |
| *Mann-Whitney test vs control* | *0.713* | | *0.721* | | *0.637* | |

**Supplemental Table 3. Measures of apical and basal dendrites of pyramidal neurons of layers II/III and V/VI and of interneurons expressing Fe65-EGFP versus batch-internal EGFP expressing neurons as control at DIV 14.**

**(A)** Pyramidal neurons.

**(B)** Multipolar interneurons.

Given is the mean ± S.E.M., and n, the number of cells analyzed. P values of Mann-Whitney rank sum test versus control are in italics. ADL, apical dendritic length (µm); BDL, mean (per cell) basal dendritic length (µm); segments, number of dendritic segments; MDL, mean dendritic length/cell; MDS, mean dendritic segments/cell; PD, number of primary dendrites.

| **Supplemental Table 3 Pyramidal cells and interneurons overexpressing Fe65 at DIV 14** | | | | | | |
| --- | --- | --- | --- | --- | --- | --- |
| A) | Pyramidal cells in layers II/III | | | Pyramidal cells in layers V/VI | | |
| Age, Condition  (Number of batches) | ADL (n)  Segments | BDL  Segments | | ADL (n)  Segments | | BDL  Segments |
| **DIV 14** Control for  Fe65 | 1676 ± 122 (32)  31.3 ± 2.4 | 358 ± 18  7.5 ± 0.5 | | 1213 ± 122 (23)  21.4 ± 2.8 | | 314 ± 36  6.7 ± 0.8 |
| Fe65 (2) | 1695 ± 165 (26)  33.9 ± 3.2 | 283 ± 21  6.2 ± 0.4 | | 1210 ± 140 (33)  20.4 ± 2.1 | | 272 ± 19  5.3 ± 0.4 |
| *Mann-Whitney test vs control* | *0.975*  *0.547* | ***0.012***  ***0.014*** | | *0.617*  *0.751* | | *0.656*  *0.206* |
|  |  |  | |  | |  |
| B) | Interneurons | | | | | |
|  | MDL (n) | | MDS | | PD | |
| **DIV 14** Control for  Fe65 | *426* ± 51 (26) | | *7.6* ± 0.8 | | *4.9* ± 0.3 | |
| Fe65 (2) | *550* ± 115 (17) | | *8.1* ± 1.5 | | *4.2* ± 0.4 | |
| *Mann-Whitney test vs control* | *0.495* | | *0.823* | | *0.165* | |

**Supplemental Figures**

**Supplemental Figure 1. Representative examples of pyramidal neurons with translucent and occluded nuclei.** Transfection with for GCaMP6m at DIV 4, imaging at DIV 12-14. **(A,B)** Cells were stained against GCaMP6m and nuclei were stained with DAPI. Cells are shown with nuclei in focus. Line scans across soma and nuclei revealed the alternating intensities (GCaMP6m in green, DAPI in blue) in healthy cell **(A)** and the higher GCaMP6m fluorescence in a cell with occluded nucleus **(B)**. **(C-F)** Calcium imaging at DIV 12-14 of GCaMP6m expressing neurons. **(C)** Cell with translucent nucleus regularly firing large amplitude calcium events. **(D)** Cell with translucent nucleus regularly firing calcium events of smaller amplitude. **(E)** Cell with occluded nucleus that fired just one large broad calcium event during the recording period. **(F)** Cell with occluded nucleus displaying small amplitude signals. Scale bar: 10 µm for **(A, B)** and 15 µm **(C-F)**.


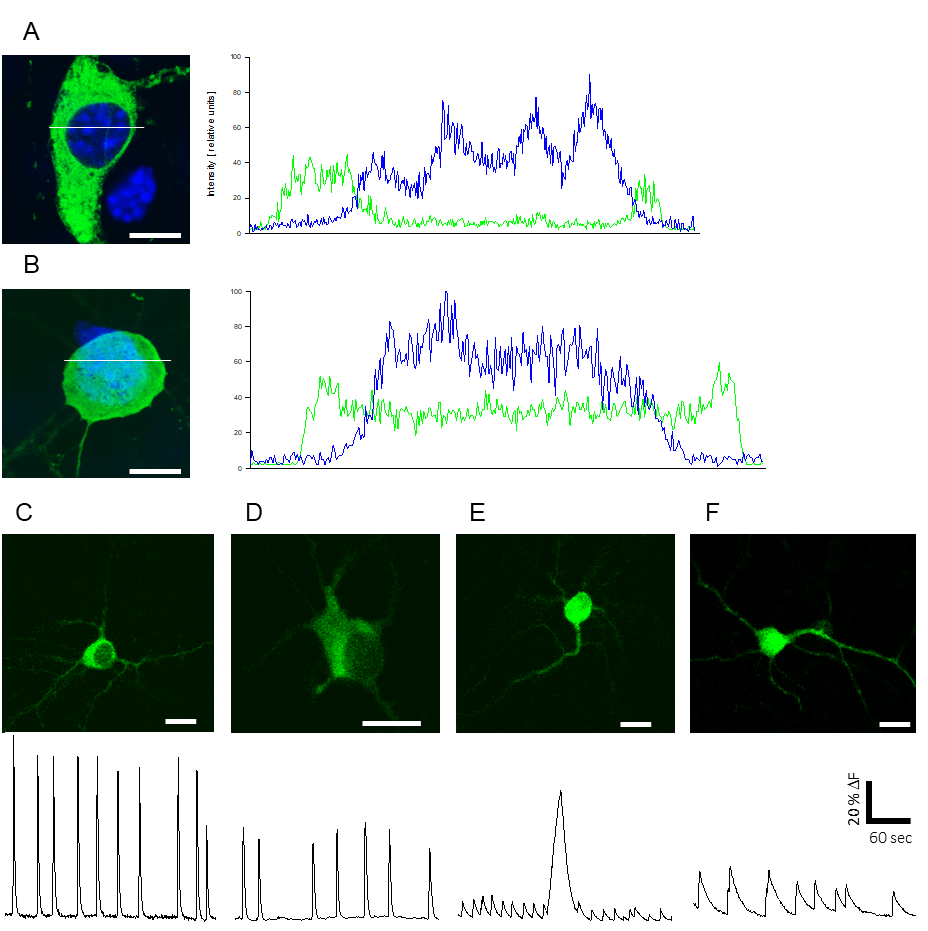


**Supplemental Figure 2. Soma size analysis of GECI expressing neurons.** **(A,B)** Neither at DIV14 nor at DIV22-25 did pyramidal cell soma sizes deviate from the control population; neurons expressing either one of the GECIs from the two laminar compartments had been pooled. **(C)** At DIV14 interneurons transfected with GCaMP3 had smaller somata compared to control EGFP interneurons. **(D)** At DIV22-25 there was no difference between GECI transfected cells versus control. ANOVA on ranks versus control was performed. Plotted is the mean ± S.E.M; the number of neurons assessed is given in the bars.

**Supplemental Figure 3. Fe65 expression in DIV 14 cortical neurons.**

(**A**) Live imaging of Fe65-EGFP/mCherry cotransfected neurons from 2 OTC at 12 and 24 h post transfection reveals that many, except nucleus “d” displayed green nuclear spheres (white arrow heads). Scale bar: 50 µm for the low magnification photomicrographs; boxed are the neurons the nuclei of which are presented in enlarged photos.

(**B**) mCherry immunoperoxidase-stained control (left) and Fe65-EGFP expressing (right) pyramidal neurons at low and higher magnification shown exemplarily to document the quality of staining used for reconstruction. Scale bar: 50 µm for low and 10 µm for higher magnification.

(**C**) Box plots of apical and basal dendritic length and segment numbers of pyramidal neurons of layers II/III, the n is given in the boxes, Mann-Whitney rank sum test versus control.

(**D**) Sholl analysis of supragranular pyramidal cells confirms the deficit in basal dendritic branching; there are less branches within about 70-100 µm distance from the soma. Insets show total dendritic branching, which is also reduced for basal dendrites. See Supplemental Table 3 for the average values of supra- and infragranular pyramidal neurons and interneurons.


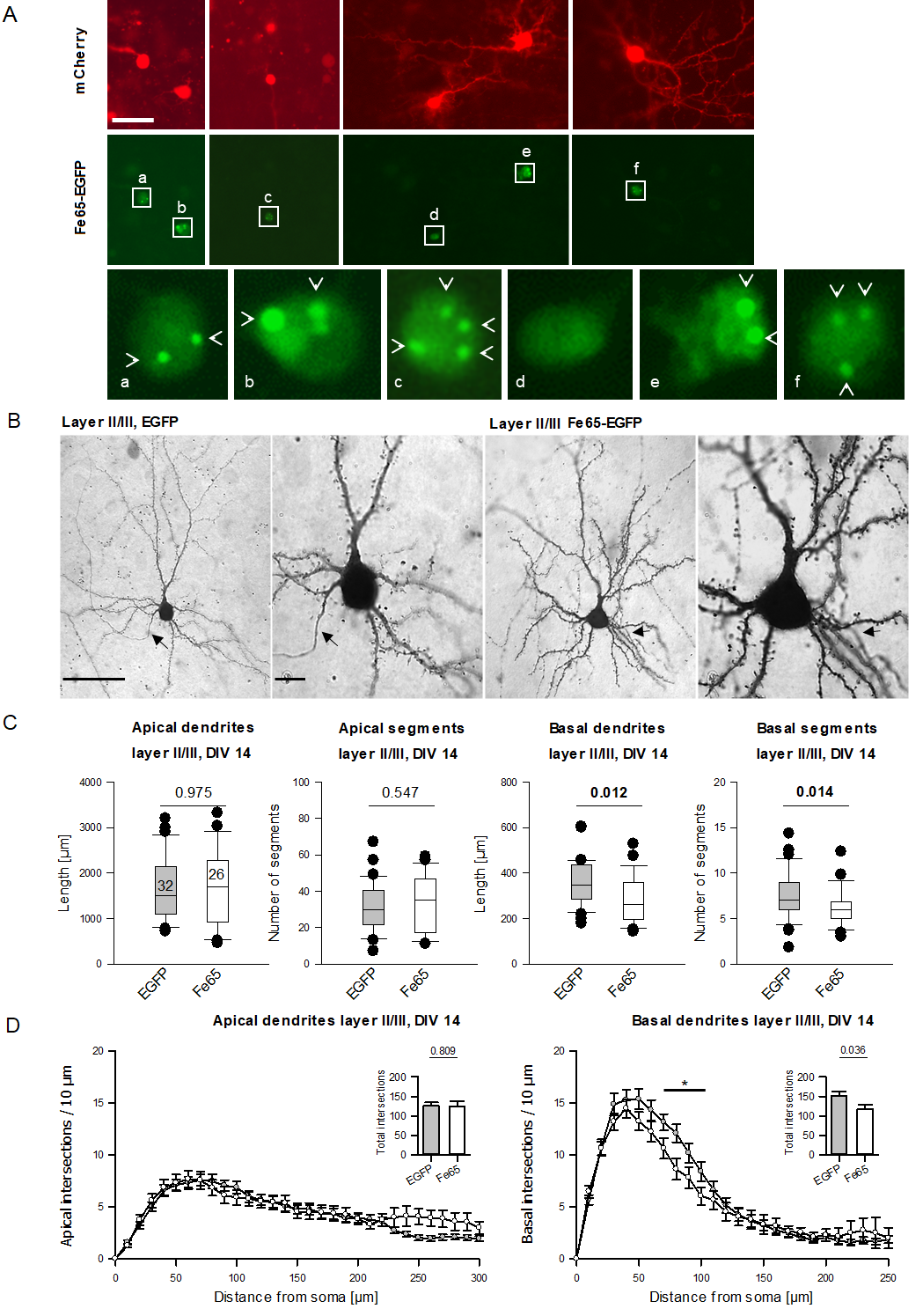

Supplement: Supplementary file 1 [file Data_Sheet_1.docx]
